# Sustainable Secondary-Raw Materials, Natural Substances and Eco-Friendly Nanomaterial-Based Approaches for Improved Surface Performances: An Overview of What They Are and How They Work

**DOI:** 10.3390/ijms24065472

**Published:** 2023-03-13

**Authors:** Silvia Sfameni, Giulia Rando, Maria Rosaria Plutino

**Affiliations:** 1 Institute for the Study of Nanostructured Materials, ISMN—CNR, Palermo, c/o Department of ChiBioFarAm, University of Messina, 98166 Messina, Italy; 2 Department of Chemical, Biological, Pharmaceutical and Environmental Sciences (ChiBioFarAm), University of Messina, 98166 Messina, Italy

**Keywords:** eco-friendly finishings, sustainability, antifouling, hydrophobic, fouling-release, smart coatings, nanomaterials, hybrid materials, sol–gel technique, natural agents, circular economy

## Abstract

To meet modern society’s requirements for sustainability and environmental protection, innovative and smart surface coatings are continually being developed to improve or impart surface functional qualities and protective features. These needs regard numerous different sectors, such as cultural heritage, building, naval, automotive, environmental remediation and textiles. In this regard, researchers and nanotechnology are therefore mostly devoted to the development of new and smart nanostructured finishings and coatings featuring different implemented properties, such as anti-vegetative or antibacterial, hydrophobic, anti-stain, fire retardant, controlled release of drugs, detection of molecules and mechanical resistance. A variety of chemical synthesis techniques are usually employed to obtain novel nanostructured materials based on the use of an appropriate polymeric matrix in combination with either functional doping molecules or blended polymers, as well as multicomponent functional precursors and nanofillers. Further efforts are being made, as described in this review, to carry out green and eco-friendly synthetic protocols, such as sol–gel synthesis, starting from bio-based, natural or waste substances, in order to produce more sustainable (multi)functional hybrid or nanocomposite coatings, with a focus on their life cycle in accordance with the circular economy principles.

## 1. Introduction

**Figure 1:**
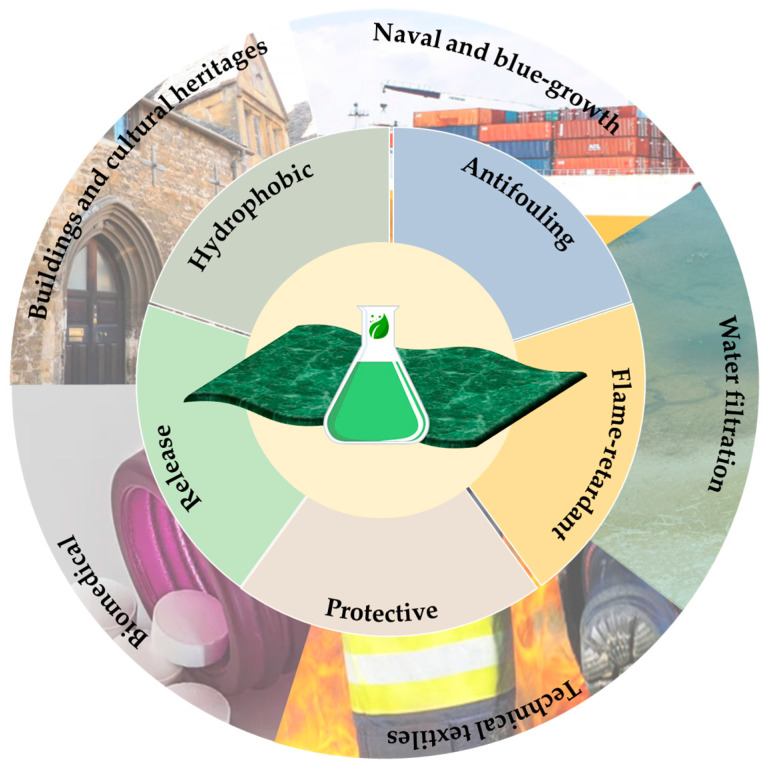
Functional and sustainable coating features and their potential application sectors.

In light of the growing global population and urbanization, the demands of modern civilization include the design of more efficient materials and more resistant surfaces to external agents; in this regard, it appears to be quite necessary to safeguard materials to increase their lifespan by reducing their rate of deterioration [1,2]. The continuous search for suitable mechanical improvement and protective agents in commonly used surfaces and items is driving nanotechnology research to develop new nanostructured coatings for various surfaces featuring enhanced properties [3,4,5]. As a matter of fact, through multidisciplinary feedback, and thanks to the great knowledge of nanotechnology, the design and development of (multi)functional nanohybrids and nanocomposites could greatly improve human daily life and well-being. Particularly, innovative Hybrid Organic-Inorganic Materials (HOIM) or rationally designed functional polymeric blends may be developed and applied in different potential application fields, such as opto-electronic [6], textiles [7], blue growth [8], building [9], mechanical [10], energy conversion [11] and storage [12], sensing [13], environmental remediation [14,15], biomedical [16,17,18] and cultural heritage sectors [19], thanks to their improved features and properties. Quite a lot of advanced nanostructured materials may be employed as surface coatings or in various composite forms for a wide range of applications due to implemented properties (i.e., antifouling [20], flame-retardant [21], drug release [22,23], sensing [24], mechanical resistance [25], and even for the absorption and degradation of environmental pollutants [26,27], as shown in Figure 1). Despite that, several approaches for the development of coatings and finishings still rely on the use of petroleum-based raw materials or doping harmful substances that are hazardous to human health and the environment, from their production cycle until their end-of-life [28]. Nevertheless, in order to achieve eco-friendly alternative functional materials and technologies, it is necessary to focus on developing sustainable manufacturing processes by employing natural, waste or organic resources; only in this way could it be possible to produce coatings that are more sustainable, recyclable, and re-usable, paying attention to their life cycles in agreement with the circular economy principles.

**Figure 2:**
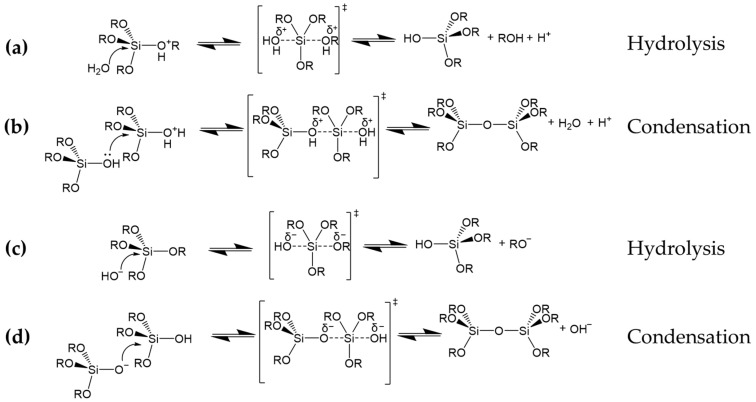
Hydrolysis and condensation reaction steps of silane alkoxide precursors occurring in acidic (**a**,**b**) and alkaline environments (**c**,**d**). Reproduced from MDPI Ref. [31].

The sol–gel technique is one of these synthetic approaches that has gained more interest lately [29]. Due to its benefits (including low process temperature, high homogeneity of the final products, absence of cytotoxicity and high versatility of the corresponding silane precursors in stable binding of functional molecules or either surface), the sol–gel approach is quite often employed for developing coating featuring stable functionalization as well as improved surface properties of different coated substrates. In this regard, a variety of synthetic pathways may be performed to produce innovative materials with opportunely tunable high molecular homogeneity and superior physical and chemical properties [30]. Sol–gel technology has been successfully applied to obtain different functional properties [7,31,32,33,34]. In particular, this procedure aims to prepare a colloidal solution (sol) in which the monomers are forced to interact with each other through subsequent hydrolysis and condensation reaction steps. These reaction steps lead to the formation of a gel, a solid and continuous lattice submerged in a liquid phase, from which the liquid can then be extracted using thermal approaches or other techniques [35]. More specifically, metallic or metalloid elements are usually employed as precursors in the sol–gel process. Organometallic precursors include silane alkoxides, which are the main fundamental elements of the majority of sol–gel processes. These molecules have a general Si(OR)_4_ formula, where R is a variable organic functional group depending on the specifically chosen alkoxide [36]. These low-cost precursors enable larger-scale applications in order to use them for procedures with improved reproducibility [37]. The hydrolysis and condensation processes, involving silane alkoxides, keep producing larger 3D frameworks with –Si–O–Si– functionalities, as obtained in both acidic (Figure 2a,b) or alkaline (Figure 2c,d) environments [38]. The flexibility of the lattice decreases as the number of –Si–O–Si– bonds rises, which causes an increase in the lattice viscosity until the latter gels [39]. The first industrial use of sol–gel technology is in thin films and coatings production, by employing different deposition approaches, including spinning [40], dipping [41], spraying [42] and doctor blading [43].

Besides the environmentally friendly sol–gel synthesis, bio-based polymers may also provide a more workable alternative to their fossil-based counterparts [44]. According to a recent market analysis by European Bioplastics, the production of bioplastics would increase globally by more than 35% between 2020 and 2025 [45]. Moreover, biomass materials have been employed as structural or common materials since ancient times, as their primary constituents were natural polymers, including lignin, cellulose, starch, chitin, and protein, which ensured sufficient strength and stability [46]. Furthermore, the large subset of naturally occurring materials, coming from agro-industrial wastes or via industrial processing of animals and agricultural products, and consisting of around five billion metric tons annually, can therefore represent a source of precious secondary raw materials for value-added and sustainable applications. For example, lignin, i.e., the second-most common biopolymer found in plant cell walls, is one of the many different types of agricultural biomasses. Large amounts of lignin are produced as a by-product of the pulp and paper industries. Environmental concerns have recently intensified efforts to employ lignin derivatives in value-added applications such as for antifouling coating preparation [47]. These eco-friendly and sustainable solutions and resources are of great interest in the field of nanotechnology. In fact, the green formulations that may be generated from these materials can be modified by adding opportune functional nanofillers, such as nanoparticles, to obtain useful protective and multifunctional coatings for various surfaces and substrates [48].

In light of these factors, the goal of this review is therefore to highlight the recent approaches for the development and characterization of hybrid materials, nanocomposites and functional coatings, also obtained through the employment of different secondary-raw, natural and eco-friendly nanostructured materials for smart, innovative and sustainable applications. In particular, coatings based on functional nanoparticles, molecules, nanofillers, sustainable recycled materials and natural substances, as dispersed appropriately in polymeric matrixes or in combination with proper sol–gel based cross-linkers and dopant agents, will be compared in order to give an overview on the recent sustainable advancement in improved surface performances materials. Furthermore, the molecular and chemical aspects behind the mechanism of action of various functional coatings are discussed to contribute to the rational safe-by-design of enhanced, green, and smart surfaces.

## 2. Functional Protective Sustainable Coatings

Protective coatings are crucial in today’s society since they may significantly improve our life, comfort, and welfare [49]. As a matter of fact, all surfaces exposed to atmospheric and environmental conditions undergo various forms of deterioration, which are influenced by a variety of causes and agents, the most notable of which are:

1. Material composition and porosity;
2. Accidents, alterations, and previous restorations or treatments;
3. Type of exposure (contaminants, climatic conditions, anthropological activities);
4. Colonization via biological or microbiological agents.

By decreasing or counteracting the deterioration kinetics, it is possible to increase the lifespan and durability of surfaces and materials by acting on the surrounding environment and defending the surface of the material itself [50,51,52].

One of the main agents from which surfaces need to be protected is water; it is the main cause of degradation, leading to a direct or indirect deterioration of materials, also due to the presence of potentially harmful solutes that can accelerate or promote this process [53]. Therefore, an ideal protective coating should exhibit different qualities like:

- Hydrophobicity;
- Transpiration capabilities towards water vapors;
- UV-resistance;
- Chemical inertness;
- Strong resistance to harmful chemical agents;
- Lead to minimal alteration of the appearance of the coated product;
- Resistance to bacterial and mold colonization.

Buildings, automobiles, textiles, industrial machinery and other infrastructure are constantly preserved to have longer life and durability due to the application of top-coatings, primers, sealants, varnishes and paints. Many of these actual surface protection treatments and coatings may still be environmentally hazardous [54,55,56]. Therefore, the main approach remains the development of unique, high-performing, ecologically friendly and functional coatings starting from bio-based formulations, waste, natural materials and compounds, as well as more sustainable processes. Some of the recent examples and approaches described and discussed in this review are collected in Table 1, reported together with a comparison of the functional agents, deposition approach, coated surfaces and implemented achieved features.

### 2.1. Anticorrosive Hybrid Nanostructured Coatings Doped with Green Corrosion Inhibitors

Every structure that is subjected to corrosion agents must be protected in order to withstand corrosive forces for the duration of the structure’s required life. Corrosion can be decreased or prevented by using specialized procedures that delay or inhibit anodic or cathodic reactions, or by eliminating conductivity between anodic and cathodic sites. Corrosion is the collective term for all chemical and electrochemical processes that return metals to their lower Gibbs free energy states. This phenomenon is largely caused by electrochemical processes, in which electrons move between two half-cell reactions [78,79,80].

Anodic or metal oxidation processes are one of the half-cell reactions that generate electrons, as shown in Equation (1), in which M represents the metal.

M^0^ → M^n+^ + ne^−^ (1)

The produced electrons are involved in the cathodic or reduction reaction, which is the other half of the cell reaction. The pH and the accessibility of different components affect cathodic reactions. Under acidic circumstances, hydrogen and oxygen reduction are the most frequent cathodic reactions, as shown in subsequent Equations (2) and (3):

2H^+^ + 2e^−^ → H_2_ (2)

O_2_ + 4H^+^ + 4e^−^ → 2H_2_O (3)

Additionally, water or oxygen reduction processes occur in neutral or alkaline conditions, as shown in the following Equations (4) and (5).

2H_2_O + 2e^−^ → H_2_ + 2OH^−^ (4)

O_2_ + 2H_2_O + 4e^−^ → 4OH^−^ (5)

The procedure for protecting the metal surface often involves two (pre)treatments that are performed in sequence, namely the preparation pretreatment of the target surface, which removes any impurities or remnants of earlier corrosive processes from the metal or substrate to be protected, and the final application of the protective top-coat [81,82]. Actual anticorrosive protective solutions require the employment of hazardous phosphating and chromating procedures, which, despite their efficacy, have considerable and negative environmental and energy consequences [83]. In particular, toxic phosphates and chromium compounds, in fact, lead to the production of large amounts of hazardous sludge that must be disposed. In this regard, new developments in the field of environmentally friendly polymer-based anticorrosive coatings of parts or specific objects/substrates have been studied and described in several research products.

**Figure 3:**
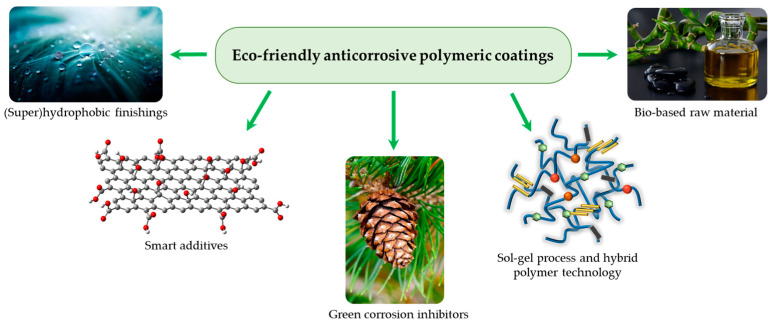
Recent trends in sustainable anticorrosive coating production. Reproduced from Ref. [85].

These cutting-edge and environmentally friendly materials/technologies, as depicted in Figure 3, can be divided into five groups: hyperbranched/hybrid polymer technologies, bio-based materials, green corrosion inhibitors, and (super)hydrophobic coatings.

The use of natural additives to enhance the properties of an anticorrosive coating is a new finding that has fascinated researchers as well as product end-users [84].

Carbon-based nanomaterials, such as graphene or graphene oxide, are of great interest due to their powerful anticorrosion feature and barrier properties, which prevent corrosive substances from entering and supplying an electron flow channel between the sacrificial anode and the substrate [86,87].

Moreover, hyperbranched polymers have most recently drawn a lot of interest in the field of polymer research due to their remarkable qualities, including high cross-linking density, superior solubility and high reactivity, thus leading to the further production of low solvent chemical-resistant and long-life coatings [88,89,90].

The application of plant extracts as environmentally benign corrosion inhibitors, such as polyphenols, has also attracted the scientific community’s interest [91]. Its inhibitory impact is due to a unique antioxidant property that causes complexes to form on the metal surface, promoting the formation of a strong barrier that protects the surface from external agents. Various studies have proved the use of pure tannins and other polyphenols as corrosion inhibitors [92,93,94].

**Figure 4:**
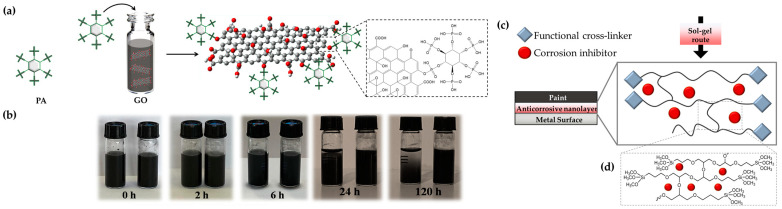
Graphene oxide intercalated phytic acid interaction (**a**) and stability of the obtained water dispersions (**b**). Sol–gel-based approach with proper silane precursors for the production of sustainable waterborne anticorrosive protective coatings (**c**) and cross-linked matrix (**d**). Reproduced from MDPI Ref. [57].

To combine the protective feature of graphene oxide and reduce its agglomeration due to π–π stacking, which makes it hard to disperse uniformly in a polymer matrix, different covalent and/or noncovalent functionalization approaches can be employed. In fact, thanks to its organic functionalities (i.e., hydroxyl, carboxyl, epoxy groups), it can react easily to form stable dispersions. An example is represented by the functionalization of graphene oxide with phytic acid, as shown in Figure 4a,b. This latter is a naturally abundant organic coordination molecule with the chemical structure of C_6_H_18_O_24_P_6_ in which each phosphate group, characterizing the molecule, is connected to a carbon atom of the cyclo-hexamehexol ring [95]. Thanks to its non-toxicity, biocompatibility and environmental friendliness, it has been widely employed in a variety of applications, i.e., food and cosmetic additives, cleaning agents, and complexation of pollutants [96,97,98]. An embedding waterborne host sol–gel matrix can be easily obtained by the use of proper silane precursors and cross-linkers, in order to produce a uniform, homogeneous, durable, stable and functional coating for metal substrates (Figure 4c,d).

In the described study, (3-glycidyloxypropyl)trimethoxysilane, hereafter indicated as GPTMS, and 3-(aminopropyl)triethoxysilane, indicated as APTES, were employed for this purpose.

The results of polarization measurements and a neutral salt spray test confirmed the anticorrosive behavior on steel and aluminum substrates of the final obtained sol–gel based nanostructured hybrid coating doped with phytic acid intercalated graphene oxide [57].

Electrochemical coatings are a popular method for protecting metallic surfaces. These coatings can be made of simple metals as well as binary and ternary alloys, which improve corrosion, oxidation, and wear resistance. An electrochemical deposition is a low-cost option for thin-film synthesis since it does not require complicated or expensive equipment and employs common raw materials. By altering deposition settings, electrodeposition enables the simple control of the coating’s chemical composition, shape, and thickness [99].

Because of its recognized anticorrosion potential and environmental friendliness, the electrodeposition of polyaniline (PANI) coatings onto metal surfaces has gained attention. The anticorrosion effect of such PANI coatings is thought to be accomplished through a variety of mechanisms, including barrier protection, corrosion inhibition, anodic protection, a shift of electrochemical reactions from the metal/coating interface to the coating/electrolyte interface, passivation, and so on. By using a potentiostatic technique, a polyaniline-reduced graphene oxide composite (PANI-rGO) film was electrochemically deposited on 5083 Al alloy. The results revealed that the presence of rGO promoted PANI electropolymerization and improved Al alloy passivation [58].

**Figure 5:**
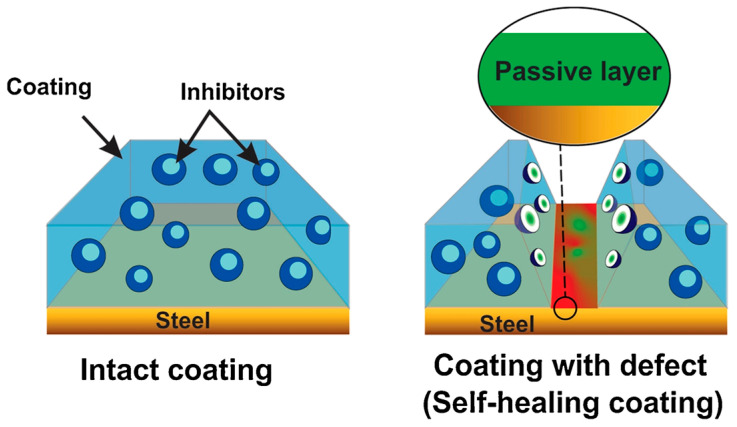
Self-healing mechanism of tannin-functionalized coating mixed with silane- hybrid ZnO nanoparticles, with passivation of the damaged steel surface by the formation of tannates. Reprinted with permission from Ref. [59]. Copyright 2019, A.F. Jaramillo, Progress in Organic Coatings, Elsevier.

As mentioned before, tannins are well-known polyphenols coming mainly from pine bark and acacia, featuring anticorrosion inhibition properties. The presence of OH groups in the ortho position of the aromatic ring of tannins leads to their reaction with iron, iron salts and oxidized steel substrates, promoting the formation of mono- and bi-ferric tannate species (tannin-Fe complexes), inhibiting the activity of the corrosion agents. In this regard, a hybrid self-healing coating, containing silane and tannins functionalized zinc oxide nanoparticles, was produced to protect steel substrates. In particular, ZnO nanoparticles, obtained by the method of arc discharge in a controlled atmosphere, were functionalized with APTES, mixed with *Pinus radiata*-derived tannin and an epoxy resin to obtain the functional hybrid epoxy. Subsequently, the obtained formulation was deposited on ASTM A36 steel plates through a spray coating approach. The anticorrosive properties of the functionalized nanoparticles were demonstrated using electrochemical analysis. Moreover, the contact angle and Kelvin probe delamination studies showed the self-healing capabilities of the film with the substrate, as shown in Figure 5 [59].

Corrosion is a more aggressive-related phenomenon affecting all the surfaces exposed in a marine environment. In this case, the proposed corrosion mechanism for steel surfaces, for example, is as follows (Equations (6)–(9)) [100]:

Fe → Fe^2+/3+^ + 2e^−^/3e^−^ (anode) (6)

O_2_ + 2H_2_O + 4e^−^ → 4OH^−^ (cathode) (7)

Fe^2+^/Fe^3+^ + 2Cl^−^/3Cl^−^ → FeCl_2_/FeCl_3_ (8)

FeCl_2_/FeCl_3_ + 2H_2_O/3H_2_O → Fe(OH)_2_/Fe(OH)_3_ + 2HCl/3HCl (unstable) (9)

**Figure 6:**
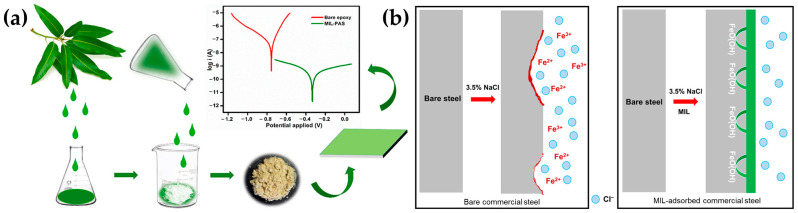
Preparation of the hybrid epoxy coating containing precipitated amorphous silica functionalized with MIL extract with its anticorrosive behavior (**a**) and mechanism of corrosion inhibition of bare commercial steel and MIL-adsorbed commercial steel in a saline environment (**b**). Adapted from Ref. [60]. Copyright 2019, K. K. Veedu, ACS Omega, American Chemical Society.

For these purposes, natural inhibitors for corrosion were also evaluated. An extract of tropical plant *Mangifera indica* L. leaf (MIL), containing a high quantity of polyphenols, was used to obtain an amorphous silica-based hybrid material, subsequently incorporated in an epoxy coating. In detail, a precipitated amorphous silica functionalized with MIL was dispersed in an epoxy resin and its polyamide curing agent with a ratio of 2:1. Finally, by a dip-coating approach, the dispersion was employed for the treatment of commercial steel substrates (Figure 6a).

The coating developed featured a superior corrosion inhibition performance of 99%, according to electrochemical measurements. The mechanism of action is explained by the formation of a FeO(OH) passive layer and an insoluble stable organometallic complex due to the hydroxylic and carboxylic functionalities of polyphenols in MIL extract preventing the Cl^−^ attack, as shown in Figure 6b and described in Equation (10).

Fe–OH_ads_ → FeO(OH)_ads_ → Fe–MIL_ads_ + 2H_2_O (10)

The results of this study thus showed a unique, environmentally friendly, effective corrosion inhibitor for the defense of steel in saline media [60].

### 2.2. Eco-Friendly Approaches for Flame-Retardant Coatings Preparation

**Figure 7:**
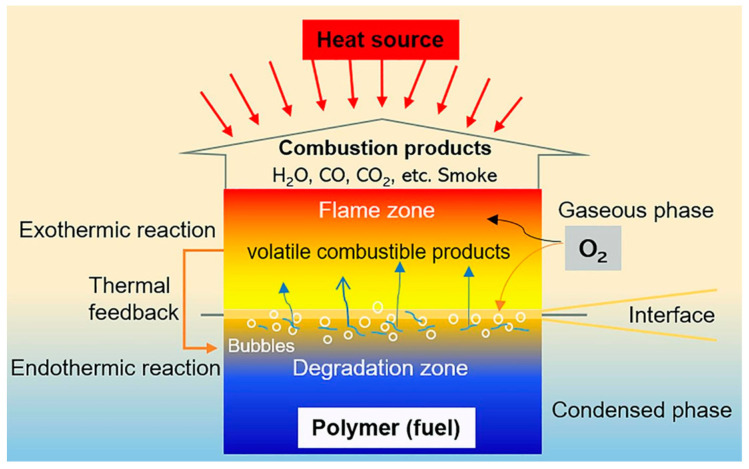
Representation of the combustion process of polymeric materials. Reprinted with permission from Ref. [102]. Copyright 2020, W. He, Progress in Materials Science, Elsevier.

The majority of polymeric materials are characterized by highly combustible chemical and organic components. However, the polymers’ inherent flammability, resulting from their chemical structures and organic components (Figure 7), limits their practical usage. The most common flame-retardants employed in various industries are mainly based on inorganic, halogenated species and phosphorus-based agents together with nitrogenous compounds (due to their synergistic effect) [101].

Flame retardants are essentially based on the trapping of free radical species involved in (H^•^ and OH^•^) oxidation reactions (gas phase) to inhibit them. Even though there are many radical reactions occurring during gas phase combustion, only the following two steps, as shown in Equations (11) and (12), are accountable for the rapid process and the energy released [103]:

H^•^ + O_2_ → OH^•^ + O^•^ (propagation stage) (11)

OH^•^ + CO → CO_2_ + H^•^ (exothermic reaction) (12)

**Figure 8:**
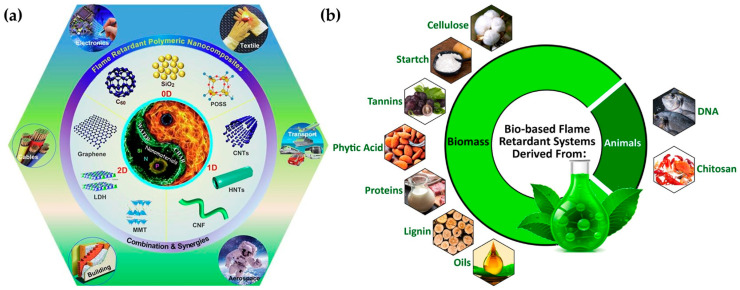
Different agents employed for the preparation of flame-retardant polymeric composites from nanomaterials (**a**) to bio-based derivatives (**b**). Reprinted with permission from Ref. [102]; copyright 2020, W. He, Progress in Materials Science, Elsevier and Ref. [104] copyright 2021, H. Vahabi, Materials Science and Engineering: R: Reports, Elsevier, respectively.

In this regard, researchers are working on the development of new flame-retardant agents and nanocomposites, employing different nanomaterials and compounds such as the ones shown in Figure 8a. The addition of a relatively modest loading level of around 5 wt% of nanomaterials to the final polymeric nanocomposites can reduce their flammability, resulting in a decrease in the heat release rate (HRR) and mass loss rate (MLR) [102]. Despite nanocomposites, naturally derived compounds can also be employed for the development of sustainable flame retardants (Figure 8b).

As a result, it is important to properly rationalize a flame-retardant finishing and consider both its physical and chemical methods of action [102,105]. Several of them, which will be further reviewed, deal with:

- Encouraging endothermic processes;
- Creating inert gases that reduce/dilute the air oxygen’s content;
- Producing an impermeable layer of protection;
- Introducing flame retardants that can scavenge and eliminate active radicals in the gas and condensed phases;
- Dehydrating, cyclizing, and cross-linking flame retardants and/or the polymer matrix to create a carbonaceous protective layer.

**Figure 9:**
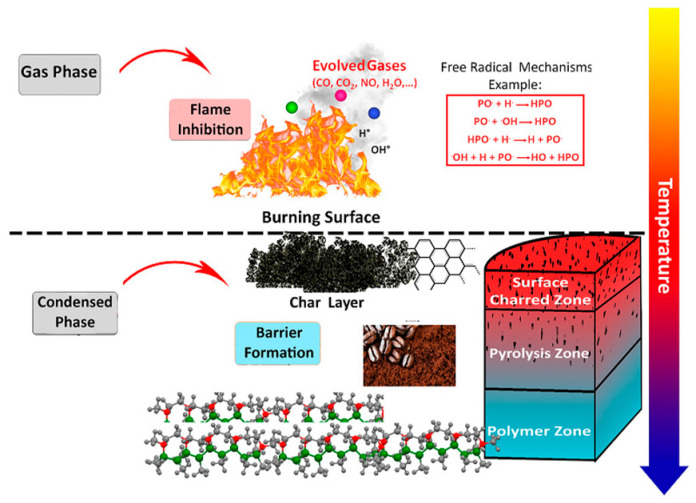
Possible mechanism of flame-retardant action of the eco-friendly composite film of epoxy resin containing spent coffee waste treated with phosphorous. Reproduced from MDPI Ref. [61].

Besides the sol–gel synthetic method [106,107], a better eco-friendly approach is represented by the use of natural or waste materials, such as coffee biowastes, as sustainable flame retardants. In particular, spent coffee grounds were chemically modified with phosphorus (dimethyl phosphite) in different ratios to obtain the P-coffee derivative. An epoxy resin containing 30 wt% P-coffee demonstrated a considerable reduction in the pHRR (40%) value by flammability analysis in pyrolysis combustion and flow calorimeter analysis. The presence of a carbon source, coffee biowaste, and phosphorus in the epoxy resin aided and sped up the production of a carbonaceous residue (char layer) that served as a barrier against heat and mass diffusion of gases into the gas phase. In the interim, the released phosphorus compounds stopped the spread of the flame by capturing free radicals in the gas phase, as shown in Figure 9 [61].

Through the in-situ synthesis of hydroxyapatite (a bio-derived polycrystalline calcium phosphate ceramic with a hexagonal structure) in the presence of lignocellulose, a novel bio-based flame retardant for poly(lactic acid) (PLA) was developed. In particular, the hydroxyapatite-modified lignocellulose was produced from a milled bagasse-derived lignocellulosic biomass and perylene dianhydride as the grafting agent. The resulting hybrid material incorporates phosphorus, hydroxyl, and aromatic functionalities to work as a long-lasting flame-retardant system. Finally, it was combined with ammonium polyphosphate and integrated into a PLA matrix to achieve higher flame retardancy than pristine PLA [108].

Among the class of cellulosic-derived products, ammonium starch phosphate carbamates (mixed starch esters primarily composed of covalently bound ammonium phosphate groups with trace quantities of carbamate groups) can be applied as new and sustainable coatings with flame-retardancy features. They are produced significantly more easily than cellulose phosphates by adopting a solvent-free eco-friendly method to combine starch with urea (as an “esterification promoter”) and phosphoric acid. Ammonia was found to be a result of ammonium starch phosphate carbamates decomposition and, as inert gas, aids in flame extinguishment [109].

Moreover, other natural derivatives and bio-macromolecules, such as proteins and casein, a phosphorylated protein derived from renewable precursors, find application as green potential flame inhibitors for textile materials due to their capacity to induce the dehydration process of cellulose chains creating a char layer rather than the depolymerization process and their ability to achieve outstanding flame retardancy action through the heat consumption process [110,111]. Additionally, precious inorganic clay compounds, such as halloysite nanotubes (HNTs) characterized by hollow nanotubular structures, are still currently used as a useful synthon for creating polymeric nanocomposite materials with implemented features due to their large surface area and low cost [112,113,114]. Different techniques have been utilized to obtain HNT nanocomposites, among which the melt mixing process seems to be the most popular. To obtain the good dispersion of HNTs into polymeric blends, the HNTs must be functionalized. The inclusion of HNTs enhanced the mechanical, thermal, and flammability qualities of the functionalized surfaces and materials [115,116].

To combine the features of casein and HNTs, a new one-dimensional nanocomposite coating was developed for different textile supports. In particular, a different number of mass loadings of HNTs (10, 30 and 50 wt%) was uniformly dispersed in a rennet casein (RC) solution prepared from renewable skim milk with the help of ultrasonication. The created bio-inspired nanocomposite was then employed to cover various textiles (cotton, polyester and blend of cotton and polyester) with the dry-pad-cure process. The coated textile fabrics’ flammability, toxic gas suppressing, reinforcing, antibacterial, and antiviral characteristics were all greatly improved. In a horizontal test, treated textile fabrics’ flame retardancy reached a rate of burning of zero, compared to 119 mm/min for untreated cotton fabrics. Further, a high LOI value of 35%, as opposed to 18.5% for a blank sample, supports the flame retardancy properties of the green coating developed [62].

### 2.3. Hydrophobic and Water-Repellent Sustainable Coatings

The use of hydrophobic surfaces in both everyday life and some industrial processes has generated a lot of interest in recent years [117].

Surface composition, as well as its texturing and roughness, have been shown to have a significant impact on its hydrophobicity. In this regard, numerous techniques, such as the deposition of layers made of nonrenewable materials (such as fluorine or hydrocarbon compounds, some wax or organic and inorganic materials), have been opportunely formulated to lower the surface energy and, therefore, improve the water contact angle (WCA) of surfaces [118,119]. In the same way, surfaces with a hierarchical structure consisting of nanostructured texturing were successful in achieving high levels of hydrophobicity and superhydrophobicity [120].

Recent attention has been focused on the design of bio-inspired surfaces by the superhydrophobicity natural model, i.e., the lotus leaf [121]. The majority of hydrophobic surfaces are produced by treatments with fluorine compounds, which have negative environmental effects due to their bioaccumulation [122]. However, the majority of the approaches that have been documented up to this point involve high costs, difficult processes, and the use of hazardous chemicals and solvents. To increase the range of industrial applications for hydrophobic surfaces with controllable morphology, it is still difficult to develop straightforward, quick, inexpensive, and environmentally responsible methods [123].

In the next paragraphs, an overview of the recent advancements in organic–inorganic hybrid and sustainable coatings is reported for the development of nanostructured finishings for cultural heritages and textile surfaces to achieve different improved features.

#### 2.3.1. Cultural Heritages Stones Protection

**Figure 10:**
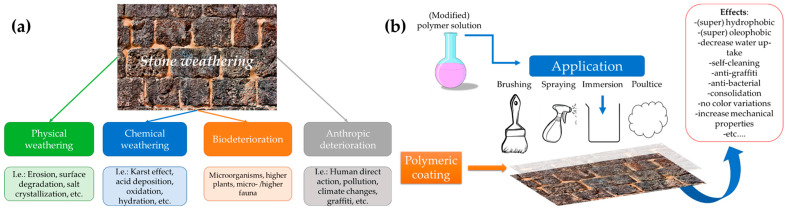
Stone weathering phenomena (**a**) and protective features and implemented properties introduced by the employing of functional polymeric formulations as coatings (**b**). Reproduced from MDPI Ref. [127].

The development of protective coatings for both transportable and immovable cultural heritage items has drawn increasingly more attention in recent years [124]. In particular, it has mostly been caused by an increased awareness of the need to preserve cultural artifacts and monuments affected by weathering exposure and the existence of reactive airborne chemicals that may interact with the materials and compromise them (Figure 10a) [125]. Several research studies have advanced significantly from the end-of-the-last-century acrylic resins to the modern biomaterials and nanoparticles used today [19,126].

The widely accepted standards of cultural heritage restoration and preservation should offer the qualities of the ideal protective coatings (including transparency, reversibility, compatibility with the surface, long lifetime, ease of synthesis, low-cost maintenance and non-toxicity) and must be typically designed for cultural objects [19].

Synthetic polymers such poly acrylates, siloxanes, and fluorinated polymers have been widely used as protective coatings for stone surfaces in cultural heritages leading to different implemented and protective features (Figure 10b).

Despite their excellent water repellent and optical clarity properties, the continuous exposure to UV light, humidity, high-temperature changes, etc., can result in their degradation, unintended cross-linking and/or chain scission reactions that diminish protection, cause yellowing, or cause the polymeric layers to separate. To improve the coating qualities and durability, silicon-based and silane-based polymers were also evaluated with and without the addition of inorganic silica (SiO_2_) and titanium dioxide (TiO_2_) nanoparticles [9,90].

A hybrid coating, made of acrylate monomers (as binding agent) and chemical compounds such as organosilanes, fluorinated silanes and titania nanoparticles, has the necessary qualities, such as thermal resistance, mechanical resistance, weathering resistance, hydrophobicity and self-cleaning, to be used as a protective coating for cultural treasures made of precious stones. In this regard, methyl methacrylate and 3-(trimethoxysilyl)propyl methacrylate were employed for the preparation of a functional acrylate-based coating. In order to increase the coating’s thermal resistance, tetraethyl orthosilicate (TEOS, an organosilane) was added to the polymeric blend. Moreover, to achieve better hydrophobicity and resilience to weathering, perfluorooctyl-trichlorosilane is further added to the mixture. Additionally, the formulation was completed by adding titanium dioxide employed to enhance the coating’s thermal resistance and photocatalytic properties. The as-obtained multifunctional organic–inorganic hybrid nanocomposite was finally employed to coat a little area of historical monuments of Persepolis in Fars province (Iran) via impregnation. Cracking resistance, hydrophobicity, water absorption resistance, weathering durability, and color durability are only a few of the qualities that have been significantly improved by the presence of opportunely added functional compounds. The hydrophobicity of the coating is improved thanks to the achievement of a rough surface obtained by the employment of the organofluorine–titania hybrid nanocomposite. Additionally, titanium dioxide enhances thermal resistance and hardness and trigger photocatalytic activity, which results in the surface’s ability to self-clean [63].

To replace fossil-derived polymers and fluorine-compounds, some other more sustainable approaches and formulations can be evaluated. An example is represented by a silane/siloxane emulsion employed as a water repellent agent coupled with chitosan and silver nitrate as biocides to create an eco-friendly finishing with both hydrophobic and biocide qualities. Chitosan is a naturally occurring amino-polysaccharide obtained from the deacetylation reaction of chitin coming from the shells of crustaceans, and it is the second-most common biopolymer in nature after cellulose. It is distinguished by a high concentration of amine and hydroxyl functionalities and has attracted growing interest in several fields [128,129,130]. The formulation Tegosivin^®^ HE 328 serves as the foundation for the protective coatings described in the example. It is an emulsion concentration built on alkoxy-functional silanes and organo-modified siloxanes. Chitosan was first added to the water repellent sol–gel formulation in various quantities as well as low concentrations of silver nitrate. A limestone called the Dom stone was finally spray-coated with the functional biocide and hydrophobic formulation achieving from 122.8° to 129.4° of WCA and a significant Chlorella vulgaris biocide impact [64].

**Figure 11:**
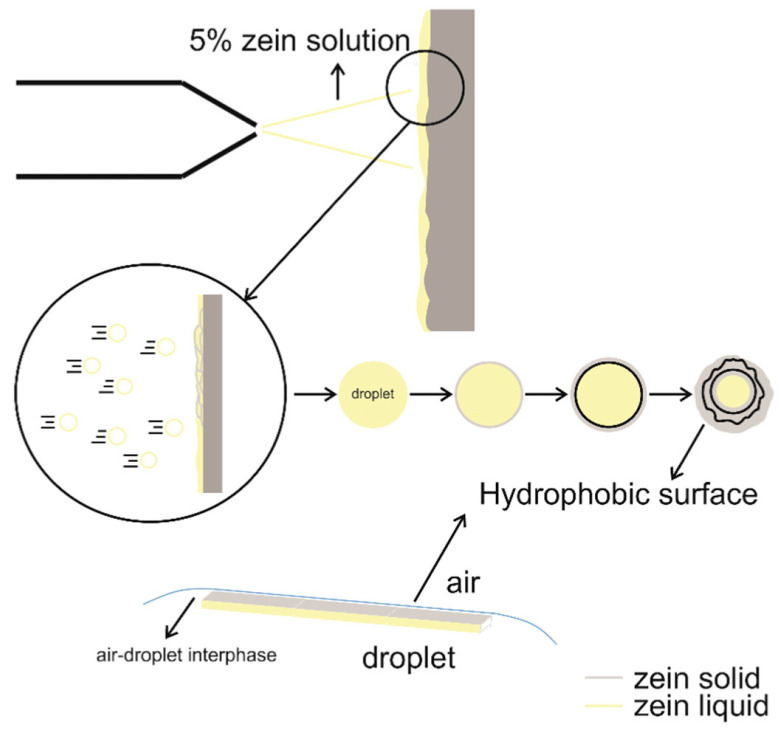
Schematization of the formation of the hydrophobic zein coating on Serena stone by the spray-coating technique. Reprinted with permission from Ref. [65]. Copyright 2021, M. Zucchelli, Progress in Organic Coatings, Elsevier.

Among the nanostructured hybrid coatings approach, some natural derivatives such as Zein can be employed to achieve hydrophobic protective coatings for cultural heritages. It is an amphiphilic prolamine obtained from corn endosperm and is characteristic of about 80% of corn proteins. Its hydrophilic behavior is determined by the relatively high content of glutamine (21–26%). A 5% (*w*/*v*) solution of zein in DMSO was therefore prepared to obtain a protective hydrophobic coating of a Serena stone (a fine-medium grain sandstone) with the spray coating technique. A WCA of about 120° was achieved. The purposed hydrophobic behavior mechanism of the obtained coating is shown in Figure 11. In particular, using spray coating, the solution was released as tiny droplets in the air, causing the solvent to quickly evaporate from the surface of the droplets. Before the solvent completely evaporated, a radial zein concentration gradient formed within each droplet in a very short amount of time. Zein began to harden, in particular, from the exterior of the droplet at the air–liquid interface toward the interior. The hydrophilic polar side of zein remains in contact with the solvent since there is still DMSO inside the droplet, forcing the non-polar hydrophobic side to face the outer portion of the droplets. As the drying process progresses, the solvent completely evaporates from the inside of the droplets as well. Atmospheric pressure placed on the partially solidified zein particles and the force of the droplets colliding with the stone surface cause the droplets to collapse on top of each other, creating the hydrophobic coating [65].

#### 2.3.2. Functionalization Approaches for Textiles Surface Modification

**Figure 12:**
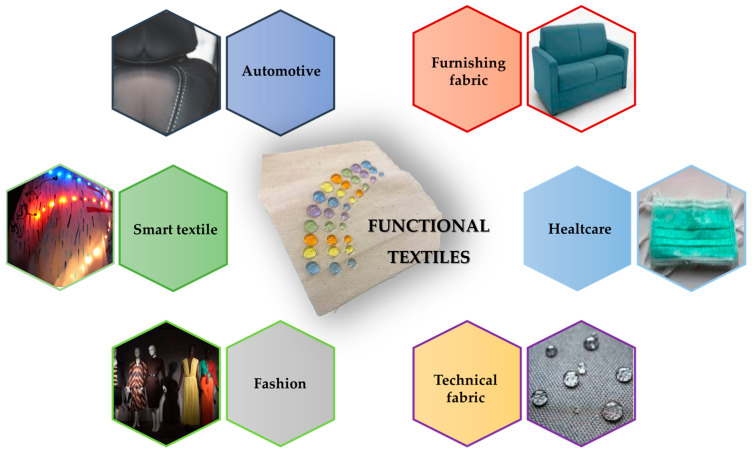
Some application fields of functional and coted textile fabrics. Reproduced from MDPI Ref. [67].

To give or enhance functional qualities of common polymer fabrics, two main approaches could be employed. The first is centered on the creation of novel fibers, which is still an expensive strategy that frequently necessitates learning new techniques for producing products and acquiring new machinery for various materials. The second strategy is based on the more useful functionalization of the surface of traditional fibers or fabrics while making minimal changes to the manufacturing methods [131]. This latter strategy is the most intriguing in the field of functional and engineered textiles. In addition to functional hi-tech fabrics, the development of more sophisticated coatings has produced engineered textiles able to interact with and react to their surroundings for applications in different innovative and nanotechnological sectors (Figure 12).

The preparation of active coatings for smart and functional textiles can be achieved using a variety of methods, most of which are traditional, while others are more modern and cutting-edge, including nanotechnologies [132,133,134]. While all of these processes are based on the attachment of certain chemical moieties to textile surfaces, they differ in terms of the modifying substance, substrate to be changed, kind of modification, and other aspects. The fabric’s wettability and the coating’s adherence to the fabric sample are both important aspects of the coating process. While the adherence of the coating is necessary for stable contact with fabric surfaces, the wettability of the textiles by the coating has a specific impact on the wicking of the treated fabrics. Intermolecular forces, ionic or covalent bonds, or weak interactions (dipole–dipole, hydrogen bond, induced dipole–dipole interactions, or dispersive) may all play a role in coating adhesion to textiles [135].

The coating process optimization must be considered since it can affect a variety of important textile qualities, such as comfort, breathability, and the hand of the coated fabric. Furthermore, the surface of the fabric and the presence of impurities can both affect the coating adhesion [136]. To achieve both functional and smart textiles with outstanding performance, uniformity and good coating distribution on the fabric surface are crucial components.

After preparing the substrate, the next step is the designed synthesis of the coating, which can be represented by a combination of functional substances in solvents or emulsions. Stabilizing additives are frequently used in this step to keep the coating solution stable for the duration of the application process. A post-processing phase may be necessary after the coating application, such as curing (cross-linking) using thermal treatment or various energy sources, including gas, an infrared oven, or UV radiation [137,138].

The sol–gel reaction, fabric impregnation process, chemical grafting, layer-by-layer (LBL) assembly, and other surface modification technologies are examples [139,140,141]. Among the aforementioned fabric finishing techniques, sol–gel technology has been widely used and adopted in a variety of industries due to its gentle reaction conditions, high efficiency, cheap cost, and environmental friendliness [142]. As a matter of fact, the sol–gel technique represents an eco-friendly method of functionalizing fabric surfaces by depositing a thin layer with specific physical properties, chemical stability and optical transparency, thus representing an advantageous technology over the ones currently available for the introduction of specific functionality onto textile fabrics. In fact, the sol–gel method makes it feasible to create textile coatings with different implemented properties [57,143,144,145,146,147,148].

**Figure 13:**
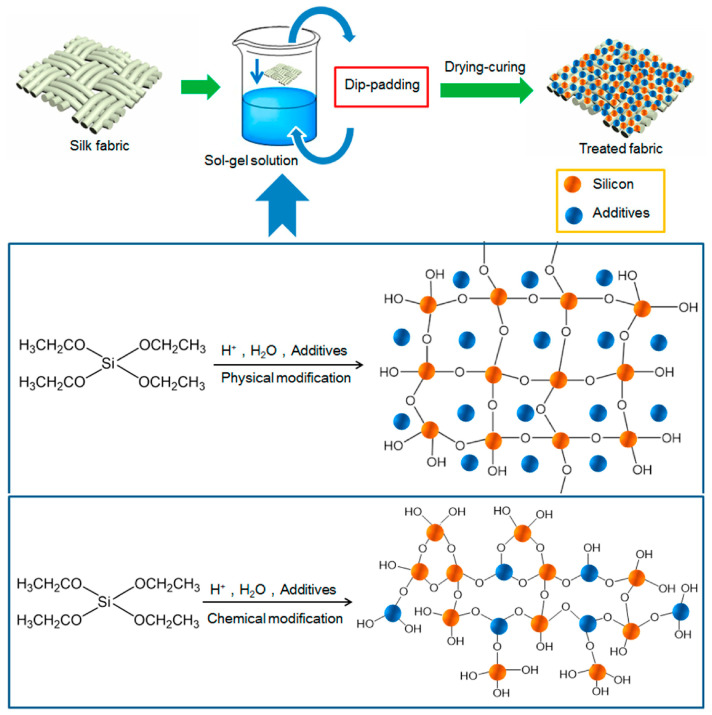
Schematic approach of sol–gel dip-padding to functionalize textile fabrics. Reproduced from MDPI Ref. [34].

The widely used sol–gel coating application procedures are based on dip-coating, padding, or spraying, producing smart or functional textiles with precisely designed properties. The dip-pad-cure method was shown to be the most popular due to its simplicity and viability from an economic standpoint. Using a padder, the fabric is immersed or dipped into a coating material solution while moving at a constant speed. After drying and curing, the process is repeated (Figure 13).

The types of chemical bonds, involving the adhesion of a coating to a surface, include covalent bonds (such as those between a silane end and an OH-group belonging to a cotton cellulose molecule), molecular attractions (such as Van der Waals forces, hydrogen bonds, dipole–dipole interactions) and, finally, the retention of the molecule by the substrate through adhesive and cohesive forces between the molecule and the substrate, as well as the molecule to itself [103,149].

Recent years have seen the application of hydrophobic treatment to textile surfaces for antifouling, self-cleaning, anti-ice, and oil–water separation purposes [150,151]. Additionally, due to their low surface energy and oil/water repellent qualities, stain-resistant surfaces and anti-stain coatings have important applications in a variety of industries, including textiles, construction, cars, and electronics [152]. Unfortunately, fluoroalkyl silanes and fluorine compounds were frequently used to further increase the surface water repellency of textile fabrics [153,154]. The ECHA committees most recently proposed restricting the use of particular perfluoroalkyl compounds (PFAS) in certain application sectors [155]. As a result, examples of environmentally friendly, fluorine-free textile finishings with stain- and water-repellent properties are also documented [156,157,158].

For example, some TEOS and citric acid superhydrophobic coatings were developed using a sol–gel approach and a spray coating/dry-curing technique to achieve fabrics (90% cotton and 10% polyester) with a WCA above 150°. The strength of adhesion between the silica and the cotton was improved by the use of citric acid [66].

**Figure 14:**
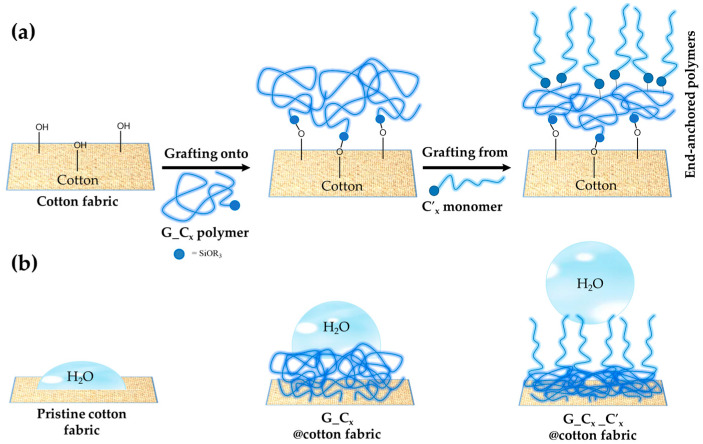
Cotton fabrics are functionalized in two steps with an alkyl(trialkoxy)silane polymer shell using covalent “grafting to” or “grafting from” procedures (**a**). The coated cotton fabrics’ surface hydrophobicity (**b**). Reproduced from MDPI Ref. [67].

Moreover, functional alkyl(trialkoxy)silane-modified hybrid nanostructured materials were successfully produced and used as hydrophobic and water-based strain resistance coatings for cotton fabrics, via the sol–gel process and pad-dry-cure technique (Figure 14a,b).

The specific objective of the reported example was to investigate different functional alkyl(trialkoxy)silanes (Hexadecyltrimethoxysilane, Triethoxy(octyl)silane and Triethoxy(ethyl)silane: C_x_ and C_y_) as precursors to synthesize efficient and stable hybrid sol–gel GPTMS-based coatings and further reduce cotton surface energy to enhance textile hydrophobicity and water-based stain resistance. By pad-dry-cure deposition of the produced nano-hybrid coatings, the double coating synthetic approach was successfully applied to increase the cotton surface’s hydrophobicity.

**Figure 15:**
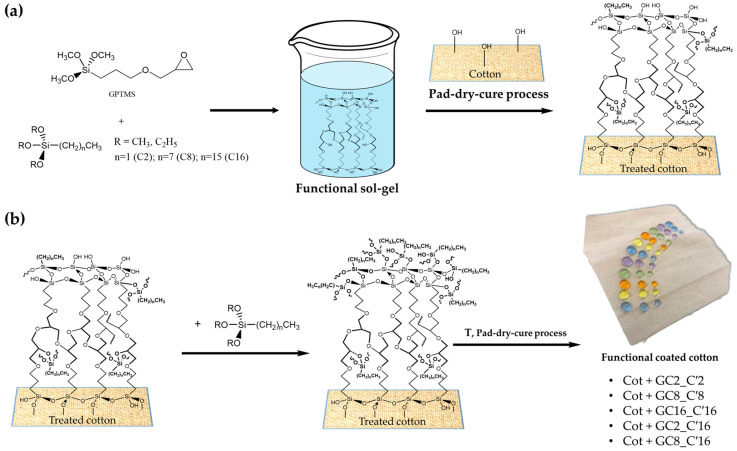
The condensation process between the cellulose and alkoxysilane ends (**a**), followed by the anchoring of alkyl(trialkoxy)silanes (**b**). Reproduced from MDPI Ref. [67].

The hydrophobicity of the fabrics was evaluated by WCA measurements, which showed that the treated fabrics have high static contact angle values (up to roughly 150°). The resistance of the treated fabric to water-based stains against several tested liquids, solutions, and soil was also proved. The moisture adsorption analysis and the air permeability test were also used to assess the fabric quality, and the results of these tests revealed that coated cotton fabrics had better overall breathability compared to pristine cotton ones. According to all experimental results, the functional alkyl(trialkoxy)silane used in the sol–gel nanohybrid coatings provided treated cotton fabrics with excellent hydrophobicity and, as a result, water repellency through the synergistic action of the surfaces’ rough structure and low surface energy according to the mechanism purposed in Figure 15.

Among sol–gel processes and silane-compounds, other sustainable approaches and polymers have been recently employed for the production of hydrophobic and multifunctional coatings for textile materials.

Polyurethanes (PUs) are a type of versatile polymer that may easily have their structure and morphology adjusted to display a wide range of mechanical, physical, chemical, and biological characteristics. Foams, thin films, textiles, technology, automotives and buildings are just a few of the industrial uses for PUs [159]. In addition to offering advantages in terms of superior material properties (such as versatility, stretchability, elasticity, and mechanical strength), processability (such as 3D printing, inkjet printing, screen printing, spray coating, and molding), and sustainability, water-based polyurethanes are constantly developed in order to replace common solvent-based polyurethanes, but also other fossil-derived polymers [160].

An example is represented by a nanocomposite coating made of single-walled carbon nanotubes (SWCNT) and a series of waterborne polyurethane-urea dispersions (WPUD) free from tin catalysts and harmful solvents. The obtained WPUD are made of 100% bio-based semi-crystalline polyester polyol (Priplast 3294) and isophorone diisocyanate and are characterized by a bio-based content ranging from 59% to 72%. The functional WPUD coating with 0.1% of SWCNT was applied by knife-coating on polyester fabrics, obtaining a hydrophobic and conductive surface [68].

**Figure 16:**
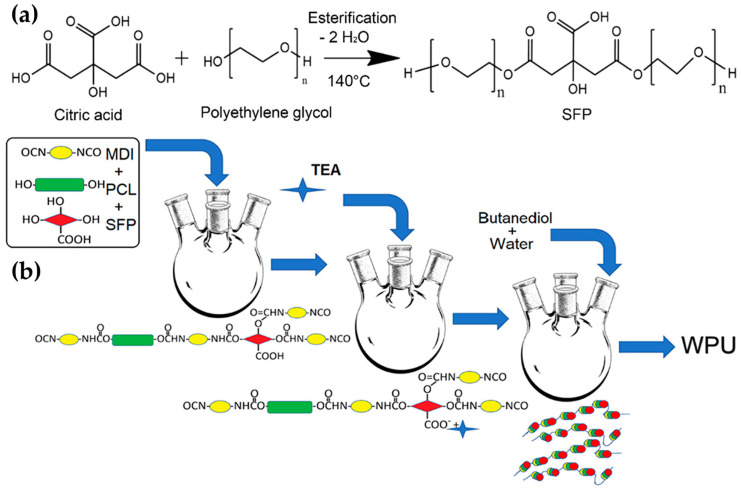
Schematic preparation of the SFP from CTA and PEG200 (**a**) and subsequent synthesis of the waterborne polyurethane (**b**) (MDI = 4,4′-methylenebisphenyl diisocyanate; PCL = polycaprolactone diol 2000; TEA = triethylamine). Reprinted with permission Ref. [69]. Copyright 2019, I. Bramhecha, Ind. Eng. Chem. Res., American Chemical Society.

Citric acid (CTA), a bio-sourced acid, and polyethylene glycol (PEG200), a polyglycol, were combined in another example to create a sustainable functional polyol (SFP) (Figure 16a) employed for the production of a waterborne polyurethane coating (Figure 16b) for cotton fabrics in order to improve the antibacterial and breathable waterproof features. The waterborne polyurethane-coated samples obtained with a knife and the roller coating approach revealed inherent antibacterial capabilities ranging from 84% to 99% against *Escherichia coli* germs and water barrier qualities without a compromise in the mechanical properties of the cotton fabric [69].

### 2.4. Anti-Fouling and Fouling-Release Sustainable Coatings

The phenomenon of fouling occurs when macromolecules, bacteria or suspended particles stick to the surface of different materials. The construction industry, cultural heritage, marine industry and industrial sectors are all interested in this problem [161,162]. In this regard, one critical use of nanostructured coatings might result in the inhibition of microbial proliferation on various surfaces. Due to the variety of fouling organisms and their adhesion processes, designing antifouling (AF) coatings has proven to be a considerable challenge over time. Significant research on biocidal and non-biocidal coatings that prevent and decrease biofouling was sparked by the need to find answers to these problems [163,164]. Most control measures in the middle of the 19th century employed paints that included biocides. However, the biocide is a highly harmful agent (similar to the now-banned tributyltin) that is put into paints as an additive and, when exposed to seawater, is gradually released into the marine environment as a result of chemical and physical phenomena [165].

**Figure 17:**
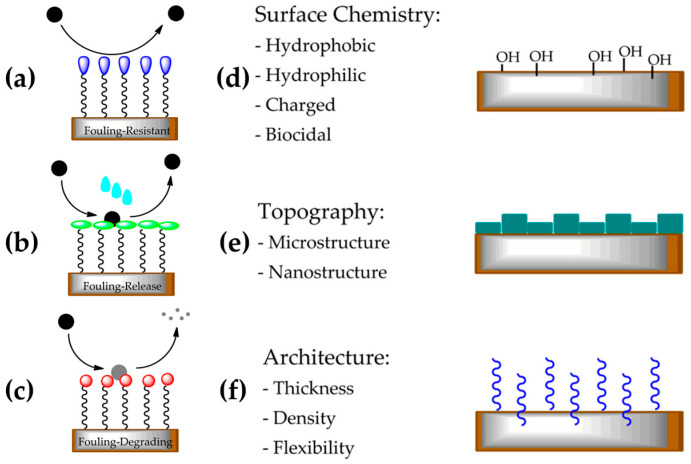
The three main antifouling strategies are shown schematically as preventing foulants from adhering to the surface (fouling-resistant) (**a**), reducing foulant interaction with the surface (fouling-release) (**b**), and degrading or eliminating bio-foulants (fouling-degrading) (**c**). Methods for giving a surface antifouling characteristics, including changing the surface chemistry (**d**), changing the surface topography (**e**), changing the coating design (**f**). Reproduced from MDPI Ref. [167].

Fouling is influenced by surface characteristics, such as surface energy and wettability. As a result, altering the surface morphology and functionalities offers a key method for providing antifouling features on different surfaces [166]. One efficient way to do this is to treat the substrate with a formulation made of antifouling and antibacterial polymers. These coatings and paints can prevent biofouling and biocorrosion because they incorporate functional anti-adhesion, antimicrobial and anticorrosion chains rather than releasing biocides (Figure 17a–f).

**Figure 18:**
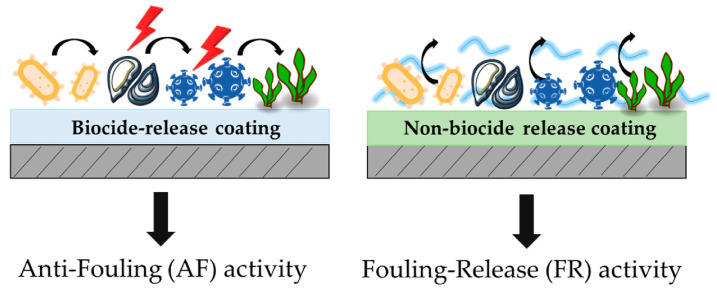
Antifouling and fouling-release activity of biocide-release and non-biocide-release coatings, respectively. Reproduced from MDPI Ref. [70].

In the next paragraphs, two main approaches of antifouling (AF) and fouling release (FR) (Figure 18) coating preparation will be explored, including the change in surface topography/hydrophilicity and the use of eco-friendly biocidal agents.

#### 2.4.1. Biocide-Free FR Sustainable Coatings

All surfaces and equipment that operate in marine environments (such as small or large boats, pylons, mining platforms, and underwater monitoring systems) are regularly exposed to the corrosive action of environmental and biological agents, referred as marine fouling [168]. Sol–gel coating technology is probably one of the most pertinent tools for the development of environmentally friendly antifouling (AF) and fouling release (FR) formulations, given the current social expectation that new clean, flexible, and effective solutions will be adopted rather than the previously employed harmful ones. Due to several limitations of utilizing biocides in antifouling coatings, numerous techniques have been developed to obtain innovative coatings designed to directly interfere with microbe adherence as a result of topography or surface chemistry changes, thus showing FR properties.

As a matter of fact, by mixing silicones with fluoropolymers, the critical surface tension can be reduced. Additionally, extensive research has been conducted on developing hydrophobic surfaces that can prevent the onset of microfouling settlements [169]. Fluoropolymers can be successfully used to create anti-adhesion surfaces due to exposed CF_2_ and CF_3_ groups at the interface, which can limit the fouling attachment depending on the degree of fluorine atom mobility [170]. These fluorinated coatings provide good antibacterial action against a variety of microorganisms and negligible adhesive properties. Furthermore, tests on microbial cells showed that the substance discharged in the liquid medium had no biocidal effects. This non-biocidal approach is mostly suitable for vessels that travel at moderately high speeds and are not idle for extended periods of time, notwithstanding the high costs of fluoropolymer-modified finishings.

On this regard, four different silane precursors were used to combine the chemical-physical properties of co-monomers containing functional fluorinated organic compounds (i.e., 3,3,3-trifluoropropyl-trimethoxysilane, F3, and glycidyl-2,2,3,3,4,4,5,5,6,6,7,7,8,8,9,9-hexadecafluorononylether, F16) with those of alkoxysilane cross-linkers subunits containing epoxy and amine groups (i.e., GPTMS and APTES) to obtain a biocide-free FR and hydrophobic coating for the marine environment. Moreover, an asymmetric nanostructured hyperbranched polymeric film was produced by mixing both F3 and F16 co-monomers, i.e., bearing both long and short per-fluorinated chains [70].

**Figure 19:**
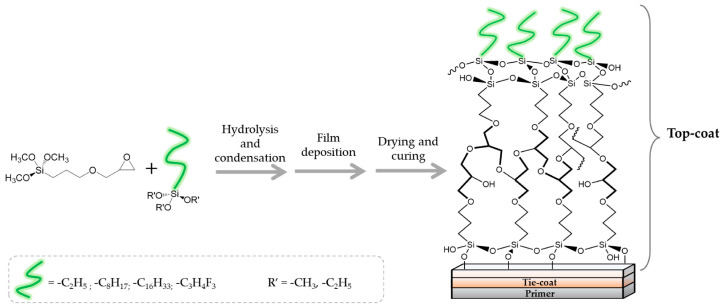
Synthetic steps involving the production of the eco-friendly sol–gel hydrophobic antifouling coating. Reproduced from MDPI Ref. [71].

For example, different painting/coating cycles are often employed in the naval sector surfaces in order to ensure adequate antifouling protection; they involve the use of three layers of polymeric AF/FR protective formulations on the hull surface exposed to the marine surrounding and are specifically indicated as primer, tie coat, and final topcoat. In another study, a research study was carried out on the design and development of functional hybrid coatings (top-coats) based on sol–gel approaches and featuring strong hydrophobic and antifouling/fouling-release capabilities. FR alternative systems were widely utilized in the biofouling mitigation treatment of various surfaces, offering a non-toxic substitute to AF coatings based on biocide- and fluorine-containing formulations. In more detail, the sol–gel cross-linker GPTMS precursor was used in conjunction with long alkyl-chain alkoxysilanes with various hydrocarbon chain lengths and hydro-repellent features (Figure 19).

This investigation showed that the surface wettability was significantly affected by these opportune organic silanes bearing suitable functional groups, such as the long alkyl chains. In addition, the coating’s hydrophobic properties were improved by the application of a commercial tie-coat layer between the primer and the topcoat, which decreased surface energy and raised the contact angle value. As a result, these eco-friendly coatings opened up the way to the development of fouling release sol–gel-based paints. By lowering the adhesion force between the fouler and the material used to construct the ship surface, their application could enable the fouling to be removed only by the movement of the ship during navigation [71].

A lotus leaf-inspired superhydrophobic coating was developed to fight microbiologically influenced corrosion in seawater environments. Copper surfaces were coated by a simple electrodeposition strategy based on a coordination-deposition process of a matrix made of an Fe-myristic acid compound from an ethanol solution in a single step. Therefore, a porous structure was developed starting from these hydrophobic compounds, which showed a dependent link between wettability and electrodeposition parameters such as electrodeposition potential and time. Due to the restricted anchoring sites and low surface energy, the as-obtained superhydrophobic matrix is verified to prevent representative diatoms and sulfate-reducing bacteria (SRB) from growing onto metal surfaces. In severe SRB suspension, the superhydrophobic matrix demonstrates remarkable corrosion resistance, suggesting a key role in combating microbiologically driven corrosion in saltwater environments [72].

**Figure 20:**
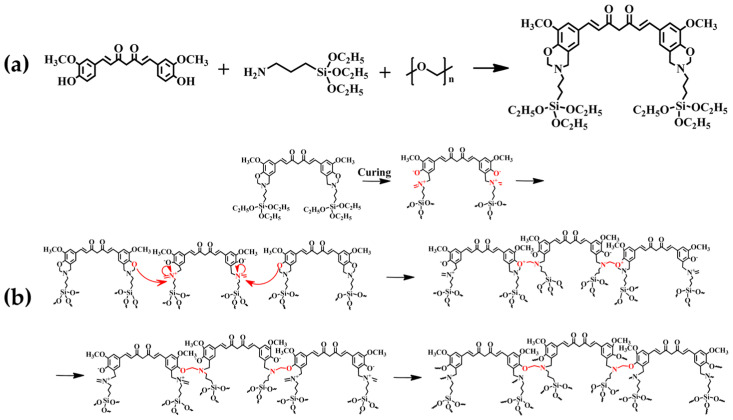
Curcumin-based benzoxazine monomer synthesis reaction (**a**) and synthetic pathway for the curcumin-based benzoxazine polymerization through thermal induction of ring opening (**b**). Reprinted with permission from Ref. [73]. Copyright 2021, Y. Deng, Composites Part B: Engineering, Elsevier.

AF/FR coatings can also be developed using natural precursors such as curcumin. In particular, a bio-based antifouling and anticorrosion benzoxazine double-layer coating for metal surfaces was obtained from the reaction of curcumin, APTES and paraformaldehyde (Figure 20a). The synthesized benzoxazine was loaded with various amounts of hydrophilic PEG molecules to modify the resin framework, give the resin its intrinsic characteristics and improve anti-biofouling performance. Curcumin has several inherent properties; moreover, the two phenolic hydroxyl groups characterizing the molecule can increase the cross-link density of the bioactive bisbenzoxazine resin (Figure 20b). Furthermore, APTES is utilized as a common and inexpensive amine source to enhance the resin cross-linking (Si-O-Si framework formation). The resins exhibited good repellent properties (prevention of settlement, adhesion, and growth of microorganisms) even in the absence of booster biocides, thanks to the advantageous effects of specific PEG content [73].

**Figure 21:**
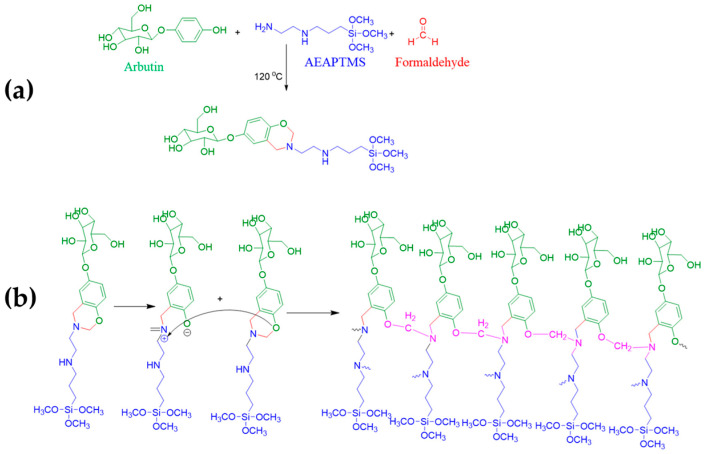
Arbutin-based benzoxazine monomer synthesis reaction (**a**) and synthetic pathway for the arbutin-based benzoxazine polymerization (**b**). Reprinted with permission from Ref. [74]. Copyright 2022, T. Periyasamy, Progress in Organic Coatings, Elsevier.

In another work, different bio-based polybenzoxazine/copolymer coatings, containing arbutin and silane functionalization featuring anticorrosive and anti-biofouling properties for low-carbon steel substrates, were developed. In detail, the plant-derived arbutin (β-d-glucoside of hydroquinone) was employed in synergic combination with the silane precursor [3-(2-aminoethylamino) propyl]trimethoxysilane (AEAPTMS) and paraformaldehyde for the production of the benzoxazine polymer resin (Figure 21a,b) applied by dip-coating on the selected substrates. Arbutin and its many hydroxyl groups contribute to its ability to enhance cross-linking. PEG or PEG-based copolymers are also added to increase surface resistance to antimicrobial adhesion. In this regard, polydopamine-PEG coating seemed to work as both an anti-bio adhesive and an anti-biofouling agent, reducing biofouling attachment on a variety of surfaces [74].

#### 2.4.2. Eco-Friendly Biocide AF Coatings

**Figure 22:**
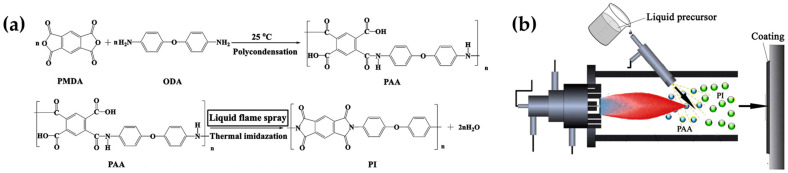
Synthetic pathway of polyimide (**a**) by liquid flame spray approach (**b**). Reprinted with permission from Ref. [75]. Copyright 2017, Y. Liu, Materials & Design, Elsevier.

Biocide-containing AF coatings are also developed by following sustainable procedures and approaches or by employing nonharmful substances for the environment and human health. In this regard, inorganic nanoparticles can find applications as biocidal agents for antifouling applications. Since the restriction on organotin chemicals, copper has become the most significant alternative biocide for antifouling applications [165]. The methods used to incorporate copper into polymeric matrices need to be further investigated to ensure the controlled release of copper ions without compromising the antifouling performances. The development of copper-containing polymer coatings using the liquid flame spray technique is an example. This technique allows the one-step preparation of polymer-encapsulated composite coatings, starting from proper formulations and dispersions containing different additives. For this purpose, copper nanoparticles were incorporated into a polyimide precursor to produce micro-structured polyimide-copper splats and coatings for the controlled release of copper and the inhibition of the tested *Bacillus* sp., *Phaeodactylum tricornutum* and *Chlorella* foulants. In particular, a Kapton-type aromatic polyimide precursor solution was obtained by mixing the monomer pyromellitic dianhydride (PMDA) and 4,4′-oxydianiline (ODA) (Figure 22a), and subsequently, the polyimide-Cu suspension was prepared by adding Cu nanoparticles (~300 nm in size). Carbon steel Q235 plates were finally coated with the liquid flame spray approach with the prepared formulation (Figure 22b) [75].

Other approaches and polymeric formulations can also be developed for the immobilization of antifouling agents such as the isocyanate-reactive Econea biocide (4-bromo-2-(4-chlorophenyl)-5-(trifluoromethyl)-1H-pyrrole-3-carbonitrile), created by functionalizing econea with a diisocyanate, to avoid their dispersion into the environment. When compared to other typical biocide-releasing coatings, this latter biocide was successfully grafted onto polydimethylsiloxane (PDMS)-based coatings, thus resulting in a tenfold decrease in the leaching qualities of the created coatings into the aquatic environment.

The multi-resistant *Staphylococcus aureus* strain was successfully inhibited by the PDMS-based coatings containing grafted Econea, which also exhibited favorable antibacterial properties and bacteriostatic activity. The coating developed on acrylic prototypes remained cleaner than the free biocide reference antifouling coating after both were submerged in Atlantic seawater for a duration of 30 months [76].

**Figure 23:**
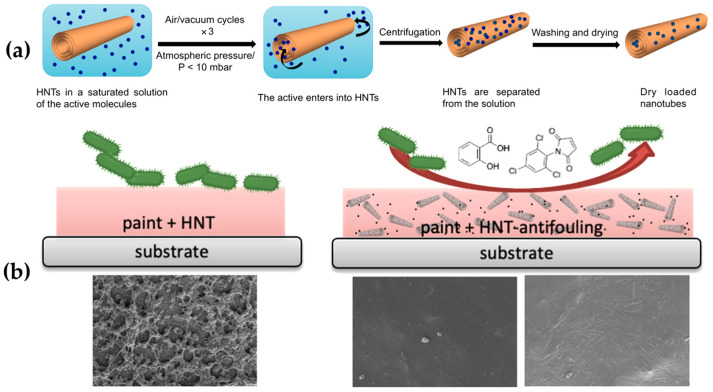
Schematic preparation of the eco-friendly sodium salicylate and N-(2,4,6-trichlorophenyl)maleimide biocide agent-loaded HNTs (**a**) and antifouling behavior of the hybrid composite epoxy-based paint obtained from the incorporation of the functional HNTs (**b**). Reprinted with permission from Ref. [77]. Copyright 2021, M. Tonelli, Colloids and Surfaces A: Physicochemical and Engineering Aspects, Elsevier.

Moreover, some nanocarriers, such as halloysite clay nanotubes, can be employed for the encapsulation and controlled slow release of biocides. Because of its tubular shape, halloysite can also be loaded with various compounds to be employed for the release of active molecules, such as antioxidants, flame retardants, corrosion inhibitors, biocides, and medicines [171,172,173]. For these purposes, two eco-sustainable antifouling compounds, sodium salicylate and N-(2,4,6-trichlorophenyl)maleimide, were successfully encapsulated into HNTs and used for the development of a hybrid composite based on epoxy paint (Figure 23a,b). 

The electrostatic interaction between the cavities of HNTs and the chosen active molecules and the solvent for their dissolution was properly tuned in order to achieve higher loading values. After the etching of HNTs, the nanotubes were loaded with the two different fillers (Figure 23a) and incorporated (10 wt% of functionalized HNT) into a commercial epoxy paint and the relative amide-based curing agent. The resulting hybrid paint was therefore poured on smooth plastic sheets with a film-coating approach. These experiments revealed that the modest and constant release of the active molecules was sufficient to stop the adhesion of *Vibrio natriegens* marine bacterium on the coated substrates (Figure 23b) [77].

## 3. Final Remarks and Future Perspectives

The growing need for more performant and long-life materials is going to have to deal with the impact on the environment of the actual technologies and approaches for the production of protective coatings and finishings. In this regard, new and more sustainable methods are constantly developed in order to reduce or replace the common fossil-derived polymers, waxes and potentially harmful compounds actually still employed for the improvement and preservation of surfaces from corrosion, fire, bacteria, water and other external and environmental degrading agents.

In this context, this review aims to give an outlook on the most recent methodologies for the eco-friendly design and development of metal, plastic, textile and stone surfaces for protective coatings and finishings. The main applications of the latter rely on the implementation of the material features, among which is their anticorrosive, flame-retardant, hydrophobic, antibacterial, antifouling and fouling-release behavior. Different synthetic protocols and approaches, such as sol–gel synthesis, are evaluated and compared. Additionally, the use of naturally derived compounds, waste materials and waterborne formulations for sustainable coatings preparation is described. Different innovative application techniques are shown to achieve a better homogeneity of the coating and nano/micro-structured textures and morphologies.

Sol–gel-based matrices, organic–inorganic hybrid materials, waterborne polyurethanes, bio-based benzoxazine, and nanocarriers for active substances are among the examples shown for achieving a solution to these challenges and thus attempting the need for less-impacting and safe coating formulations. The scientific advancements in the area of sustainable nanostructured coatings highlighted in this study show the significance of the role that nanotechnology can play in the production of increasingly high-value and competitive coatings and finishings for different application sectors. Table 2 reports a comparison between each strategy described in this review, mainly focusing on their advantages and disadvantages.

However, the development of even more advanced polymers and formulations that can provide surfaces with permanent or long-lasting multifunctional qualities still poses some difficulties. For instance, a multi-layer approach could be employed for surface preparation and functionalization. The advantages of this approach rely on a better adhesion of the final functional coating to the pre-treated surface (i.e., with proper tie-coats and primer employment) but also on a higher exposure to the active sites of the specimen’s functional coating. Other problems come from the attempts to reduce the environmental impact of the finishing procedures and to implement these eco-friendly coatings on an industrial scale with the aim to be commercialized in the reference market when applied to several materials and surfaces of everyday life. In this context, a multidisciplinary strategy is required to attain these goals. As a result, collaboration across many scientific areas and research activities involved in sustainable coating manufacturing for various applications may be a solution to these challenges, thus resulting in more performant and improved surfaces.

## Figures and Tables

**Table 1:** Some recent developments in the design and application of functional and sustainable coatings based on natural substances, waste-derived compounds, waterborne formulations and eco-friendly approaches.

Functional Precursors	Deposition Approach	Coated Surface	Properties	Ref.
GPTMS ^1^, APTES ^2^, GO ^3^ and phytic acid	Film coating	AQ-36 aluminum and QD-36 carbon steel	Anticorrosive	[57]
Polyaniline and reduced GO	Electrodeposition	5083 Al alloy	Anticorrosive	[58]
ZnO nanoparticles, APTES, *Pinus radiata*-derived tannin and epoxy resin	Spray coating	ASTM A36 steel plates	Hydrophobic, anticorrosive, self-healing	[59]
*Mangifera indica* L. leaf extract, amorphous silica and epoxy resin	Dip-coating	Carbon steel	Anticorrosive	[60]
Phosphite-treated coffee biowaste and epoxy resin	Film-casting	Glass substrate to peel off the final film	Flame retardant	[61]
Halloysite and rennet casein	Dip-pad-dry-cure	Cotton, polyester and blend of cotton and polyester	Flame retardant, antibacterial, antiviral	[62]
Methyl methacrylate, TMSM ^4^, TEOS ^5^, FOTCS ^6^ and TiO_2_	Impregnation	Stone of a historical monument	Thermal, mechanical and weathering resistance, hydrophobicity and self-cleaning	[63]
Silane/siloxane emulsion, chitosan and AgNO_3_	Spray-coating with airbrush	Building porous limestone	Water repellent and biocide	[64]
Zein solution	Spray coating	Sandstone	Water repellent	[65]
TEOS and citric acid	Spray coating/dry-curing	90% cotton and 10% polyester fabrics	Hydrophobicity	[66]
GPTMS, Hexadecyltrimethoxysilane, Triethoxy(octyl)silane and Triethoxy(ethyl)silane	Dip-pad-dry-cure	Cotton fabrics	Water repellent, water-based anti-stain	[67]
SWCNT ^7^ and WPUD ^8^	Knife-coating	Polyester fabrics	Hydrophobic and conductive	[68]
SFP ^9^, MDI ^10^ and PCL ^11^	Knife on roller coating	Cotton fabrics	Antibacterial and breathable waterproof	[69]
GPTMS, APTES, F3 ^12^ and F16 ^13^	Film coating	Glass plates	Hydrophobic and fouling-release	[70]
GPTMS and alkyl(trialkoxy)silanes	Film coating	Glass plates treated with a commercial primer and tie-coat	Hydrophobic and fouling-release	[71]
Fe-myristic acid compound	Electrodeposition	Cu metal	Superhydrophobic, bio-corrosion and bio-adhesion inhibition	[72]
Curcumin, APTES, paraformaldehyde and PEG ^14^	Film coating	Carbon steel	Anticorrosive, inhibitor of microorganisms’ settlement, adhesion and growth	[73]
Arbutin, AEAPTMS ^15^, paraformaldehyde and PEG	Dip coating	Low carbon steel	Anticorrosive and anti-biofouling adhesion	[74]
PMDA ^16^, ODA ^17^, Cu nanoparticles	Liquid flame spray	Carbon steel Q235	Biocide-release antifouling	[75]
Econea, diisocyanate and PDMS ^18^	Dip-coating	Acrylic prototypes	Antibacterial and bacteriostatic activity	[76]
Halloysite, sodium salicylate, N-(2,4,6-trichlorophenyl)maleimide and epoxy paint	Film coating	Plastic sheets	Biocide-release antifouling	[77]

^1^ GPTMS = (3-Glycidyloxypropyl)trimethoxysilane; ^2^ APTES = 3-(aminopropyl)triethoxysilane; ^3^ GO = graphene oxide; ^4^ TMSM = 3-(trimethoxysilyl)propyl methacrylate; ^5^ TEOS = tetraethyl orthosilicate; ^6^ FOCTS = perfluorooctyl-trichlorosilane; ^7^ SWCNT = single-walled carbon nanotubes; ^8^ WPUD = waterborne polyurethane-urea dispersions; ^9^ SFP = polyol made from citric acid and polyethylene glycol; ^10^ MDI = 4,4′-methylenebisphenyl diisocyanate; ^11^ PCL = polycaprolactone diol 2000; ^12^ F3 = 3,3,3-trifluoropropyl-trimethoxysilane; ^13^ F16 = glycidyl-2,2,3,3,4,4,5,5,6,6,7,7,8,8,9,9-hexadecafluorononylether; ^14^ PEG = polyethylene glycol; ^15^ AEAPTMS = [3-(2-aminoethylamino) propyl]trimethoxysilane; ^16^ PMDA = pyromellitic dianhydride; ^17^ ODA = 4,4′-oxydianiline; ^18^ PDMS = polydimethylsiloxane.

**Table 2:** Advantages and disadvantages of all reported strategies for sustainable and functional coating development.

Coating Strategy	Advantages	Disadvantages
Sol–gel	High durability Easy functionalization Easy management No cytotoxicity Versatility Uniform coating thickness	In some cases, needs of thermal curing Hard to obtain crack-free coatings Some substrates needs a pretreatment before the sol–gel deposition Low porosity
(Blended)polymers and resins	Reduction of the fossil-based polymer content High-mechanical features	Usage of a minimum of fossil-based epoxy resins and polymers
Bio-based systems	Zero fossil-based content Tunable functionalities Low processing temperatures	Starting materials and polymers have an unfavorable cost influence considering vast application scales Lower durability related to their predisposition to biodegradation
Secondary-raw materials	Recycle of waste materials Development of value-added coatings	Lower control of the homogeneity of the coating Sometimes long synthetic protocols for the treatment of the waste matrix
Eco-friendly nanomaterials	Reduced use of toxic organic solvents Improved active surface Modulated functionalities Often one-step synthesis protocols	Need for a proper design for the integration and compatibilization with the polymeric or organic/inorganic matrices Requiring a strong immobilization in the matrix to avoid their release

## Data Availability

Not applicable.
